# An inhibitor of apoptosis protein (*Es*IAP1) from Chinese mitten crab *Eriocheir sinensis* regulates apoptosis through inhibiting the activity of *Es*Caspase-3/7-1

**DOI:** 10.1038/s41598-019-56971-1

**Published:** 2019-12-31

**Authors:** Chen Qu, Jiejie Sun, Qingsong Xu, Xiaojing Lv, Wen Yang, Feifei Wang, Ying Wang, Qilin Yi, Zhihao Jia, Lingling Wang, Linsheng Song

**Affiliations:** 1 Liaoning Key Laboratory of Marine Animal Immunology, Dalian Ocean University, Dalian, 116023 China; 2 Laboratory of Marine Fisheries Science and Food Production Processes, Qingdao National Laboratory for Marine Science and Technology, Qingdao, 266235 China; 3 Liaoning Key Laboratory of Marine Animal Immunology & Disease Control, Dalian Ocean University, Dalian, 116023 China; 4 Dalian Key Laboratory of Aquatic Animal Disease Prevention and Control, Dalian Ocean University, Dalian, 116023 China

**Keywords:** Innate immunity, Transcription

## Abstract

Inhibitor of apoptosis proteins (IAPs) maintain the balance between cell proliferation and cell death by inhibiting caspase activities and mediating immune responses. In the present study, a homolog of IAP (designated as *Es*IAP1) was identified from Chinese mitten crab *Eriocheir sinensis*. *Es*IAP1 consisted of 451 amino acids containing two baculoviral IAP repeat (BIR) domains with the conserved Cx2 Cx6 Wx3 Dx5 Hx6 C motifs. *Es*IAP1 mRNA was expressed in various tissues and its expression level in hemocytes increased significantly (*p* < 0.01) at 12–48 h after lipopolysaccharide stimulation. In the hemocytes, *Es*IAP1 protein was mainly distributed in the cytoplasm. The hydrolytic activity of recombinant *Es*Caspase-3/7-1 against the substrate Ac-DEVD-*p*NA decreased after incubation with r*Es*IAP1. Moreover, r*Es*IAP1 could directly combine with r*Es*Caspase-3/7-1 *in vitro*. After *Es*IAP1 was interfered by dsRNA, the mRNA expression and the hydrolytic activity of *Es*Caspase-3/7-1 increased significantly, which was 2.26-fold (*p* < 0.05) and 1.71-fold (*p* < 0.05) compared to that in the dsGFP group, respectively. These results collectively demonstrated that *Es*IAP1 might play an important role in apoptosis pathway by regulating the activity of *Es*Caspase-3/7-1 in *E. sinensis*.

## Introduction

Apoptosis is a type of cell death which plays an important role in regulating growth, development, and immune responses^1,2^. Apoptosis is tightly controlled by multiple regulators, and the interaction between positive and negative regulators determines whether this program is activated or inhibited^3^. A family of cysteine-aspartic specific proteases known as caspases are considered as the executors of apoptosis, which cleave their substrates after the aspartate residue leading to protein degradation and apoptosis^4^. The modulation of apoptosis can be achieved by the dynamic expression of BCL-2 protein family members as well as the inhibitor of apoptosis proteins (IAPs)^5,6^.

IAP was firstly recognized in baculoviruses which could inhibit apoptosis in infected cells in 1993^7^. Subsequently, numerous IAP homologues have been identified in vertebrates, which is primarily devided into five groups including X-linked IAP (XIAP), c-IAP1, c-IAP2, NAIP, and Survivin^8^. All the IAPs contain one to three baculovirus IAP repeats (BIR) domains, which is consisted of approximately 70 amino acid residues^6^. The IAP family members differ in the number of BIR domains, and some of them also contain a RING finger domain. XIAP, c-IAP1 and c-IAP2 comprise three BIRs in the N-terminus and a RING finger in the C-terminus, while NAIP contains three BIRs without RING finger domain, and Survivin and BRUCE include only one BIR^9^. Accumulating evidences have favored that some vertebrate IAPs, such as XIAP, c-IAP1 and c-IAP2, could directly bind to the activated caspase-3 and -7, and inhibit their activities^10,11^. The BIR domains have been suggested to be responsible for the inhibition of caspases^12,13^. For instance, the BIR motifs of c-IAP1 and c-IAP2 from *Homo sapiens* were evidenced for their ability to inhibit active recombinant caspases *in vitro*^10^. The BIR2 of XIAP from *H. sapiens*, but not the BIR1 or the BIR3, was able to interact with caspase-3 and -7 with an apparent inhibition^14^. It was reported that the RING finger domain in IAPs could coordinate two zinc atoms^15^. In *H. sapiens*, the RING finger domain of XIAP could act as an E3 ubiquitin ligase^16^. Moreover, it could also recruit E2 ubiquitin-conjugating enzymes and transfer ubiquitin to its target proteins bound to IAPs^17–19^.

Recently, IAP homologues have also been discovered in various species of invertebrates. The invertebrate IAP homologues also contain one to three BIR domains, and some of them possess a RING finger domain. Some invertebrate IAPs were found to share the similar function with their homologues in vertebrates, which could play vital roles in the regulation of apoptosis and immune response against invading pathogens. For example, DIAP1 and DIAP2 were identified in fruit fly *Drosophila melanogaster* with two and three BIR domains, respectively^20,21^. DIAP2 was able to regulate the expression of antimicrobial peptides (AMPs) in response to gram-negative bacterial infection through modulating the immune deficiency (IMD) pathway in *D. melanogaster*^22,23^. *Lv*IAP1 with three BIR domains identified from shrimp *Litopenaeus vannamei* was reported to play vital roles in the regulation of shrimp hemocyte apoptosis response against white spot syndrome virus stimulation^24^. Two IAPs (named as *Cg*IAP1 and *Cg*IAP2) containing two BIR domains were characterized in pacific oyster *Crassostrea gigas*, and they were found to to be involved in regulating the apoptosis pathway and immune defense against bacterial infections^25,26^. It has been demonstrated that some IAPs could inhibit the activation of caspases in invertebrates. DIAP1 could interact with caspase DRONC and interfere its activation in *D. melanogaster*^21^. The BIR2 domain of *Cg*IAP2 could interact with the initiator caspase *Cg*Caspase-2 to participate in apoptosis inhibition^25^. The diverse caspase family members have also been discovered in various species of crustacean. For example, three caspases (*Es*Caspase-3, -7 and -8) were characterized in *E. sinensis* to play crucial roles in Cadmium-induced apoptosis^27^, and two caspases (*Es*Caspase-3/7-1 and *Es*Caspase-3-like) were involved in innate immune response under pathogen induced apoptosis^28,29^. In shrimp *L. vannamei*, there were four caspases (*Lv*Caspase-2, -3, -4 and -5) identified to play role in the host defense against white spot syndrome virus^30^. Compared with vertebrate IAPs and caspases, the knowledge about the interaction modes of the large family of invertebrate IAPs and caspases as well as their involvements in apoptosis is still quite meagre.

*E. sinensis* is one of the important economic species, and the industry of *E. sinensis* aquaculture has been increasing rapidly in China^31^. With the development of aquaculture, various diseases caused by bacteria, viruses or other pathogenic organisms have frequently occurred in cultured *E. sinensis* and caused catastrophic losses^32^. Therefore, the better understanding of immune response mechanism is helpful for controlling the diseases and reducing economic losses. In crabs, hemocytes are found to play crucial roles in defending against pathogen invasion and they can be induced to apoptosis after pathogen stimulation^33^. IAPs as inhibitors of apoptosis proteins play critical roles in inhibiting the cell apoptosis. In the present study, a novel IAP (designated as *Es*IAP1) was identified from Chinese mitten crab *E. sinensis* with the objectives (1) to investigate its mRNA distribution in tissues and its mRNA expression profile in response to immune stimulations, (2) to determine its subcellular localization in crab hemocytes, (3) to validate the interaction of r*Es*IAP1 and r*Es*Caspase-3/7-1 *in vitro*, as well as the potential regulation between *Es*IAP1 and *Es*Caspase-3/7-1 *in vivo*, hopefully to provide more information to understand the apoptosis regulation mechanism in crustaceans.

## Results

### The sequence characteristics and phylogeny of *Es*IAP1

**Figure 1:**
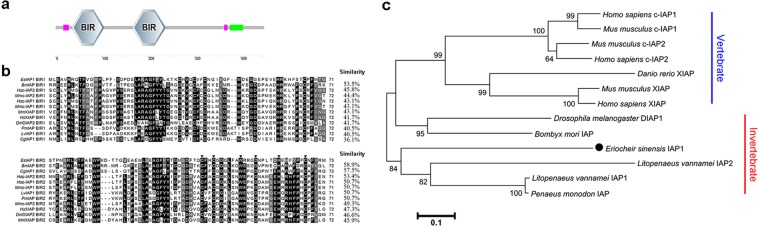
The sequence characteristics and phylogeny of *Es*IAP1. (**a**) The predicted structural domain of *Es*IAP1, which contains two BIR domains. (**b**) Multi-sequence alignment the amino acids sequences of BIR1 and BIR2 domains among IAP family members. The species and the GenBank accession numbers are as follows: *Homo sapiens* c-IAP1 (Q13490.2), *Mus musculus* c-IAP1 (Q62210.1), *H. sapiens* c-IAP2 (Q13489.2), *M. musculus* c-IAP2 (O08863.2), *M. musculus* XIAP (AAB58376.1), *H. sapiens* XIAP (AAC50373.1), *Litopenaeus vannamei* IAP1 (ADH03018.1), *Bombyx mori* IAP (NP_001037024), *Penaeus monodon* IAP (NP_001037024.), *Crassostrea gigas* IAP1 (AEB54799.1), and *Drosophila melanogaster* DIAP2 (Q24307.3). Conserved cysteine and histidine residues of *Es*IAP1 are marked with “▼”. Other conserved, but not consensus amino acids are shaded in gray. (**c**) The unrooted tree was built based on the amino acid sequences of 13 IAP family members. The species and the GenBank accession numbers were as follows: *H. sapiens* c-IAP1 (Q13490.2), *M. musculus* c-IAP1 (Q62210.1), *H. sapiens* c-IAP2 (Q13489.2), *M. musculus* c-IAP2 (O08863.2), *M. musculus* XIAP (AAB58376.1), *H. sapiens* XIAP (AAC50373.1), *L. vannamei* IAP1 (ADH03018.1), *L. vannamei* IAP2 (ADY38394.1), *P. monodon* IAP (ABO38431.1), *Danio rerio* XIAP (AAI33127.1), *B. mori* IAP (NP_001037024), *D. melanogaster* DIAP1 (Q24306.2) and *E. sinensis* IAP1 (AWK27045).

A novel sequence of *Es*IAP1 (GenBank accession numbers MF351747) was identified from *E. sinensis* genome database. The open reading frame of *Es*IAP1 was of 1,356 bp, encoding a predicted polypeptide of 451 amino acids with calculated molecular weight of approximately 50 kDa. SMART analysis demonstrated that *Es*IAP1 contained two BIR domains (BIR1 and BIR2). The conserved cysteine and histidine residues and the spacing between them in the reported BIR2 domains (Cx_2_ Cx_6_ Wx_3_ Dx_5_ Hx_6_ C) were also identified in the BIR2 domain of *Es*IAP1 (Fig. 1a). The deduced amino acid sequences of BIR1 and BIR2 domains of *Es*IAP1 shared high sequence similarities with the corresponding domains of other IAPs, such as those from *L. vannamei* IAP1 (40.5% and 50.7%), *Mus musculus* XIAP (43.1% and 45.9%), *H. sapiens* c-IAP2 (45.8% and 53.4%), *H. sapiens* XIAP (41.7% and 47.3%), *M. musculus* c-IAP2 (44.4% and 49.3%), *D. melanogaster* DIAP2 (41.7% and 46.6%), *Bombyx mori* IAP (53.5% and 58.9%), *H. sapiens* c-IAP1 (43.1% and 50.7%), *M. musculus* c-IAP1 (43.1% and 50.7%), *C. gigas* (36.1% and 57.5%), and *Penaeus monodon* IAP (40.5% and 50.7%) (Fig. 1b). To evaluate the evolutional relationship of *Es*IAP1, a phylogenetic tree was constructed based on the amino acid sequences of 13 IAP members by the neighbor-joining method. *Es*IAP1 was firstly clustered with other arthropod IAPs in the phylogenetic tree, and then grouped with invertebrate IAPs, and finally clustered into the vertebrate XIAPs and c-IAPs (Fig. 1c).

### Tissue distribution of *Es*IAP1 mRNA and subcellular localization of *Es*IAP1 in hemocytes

**Figure 2:**
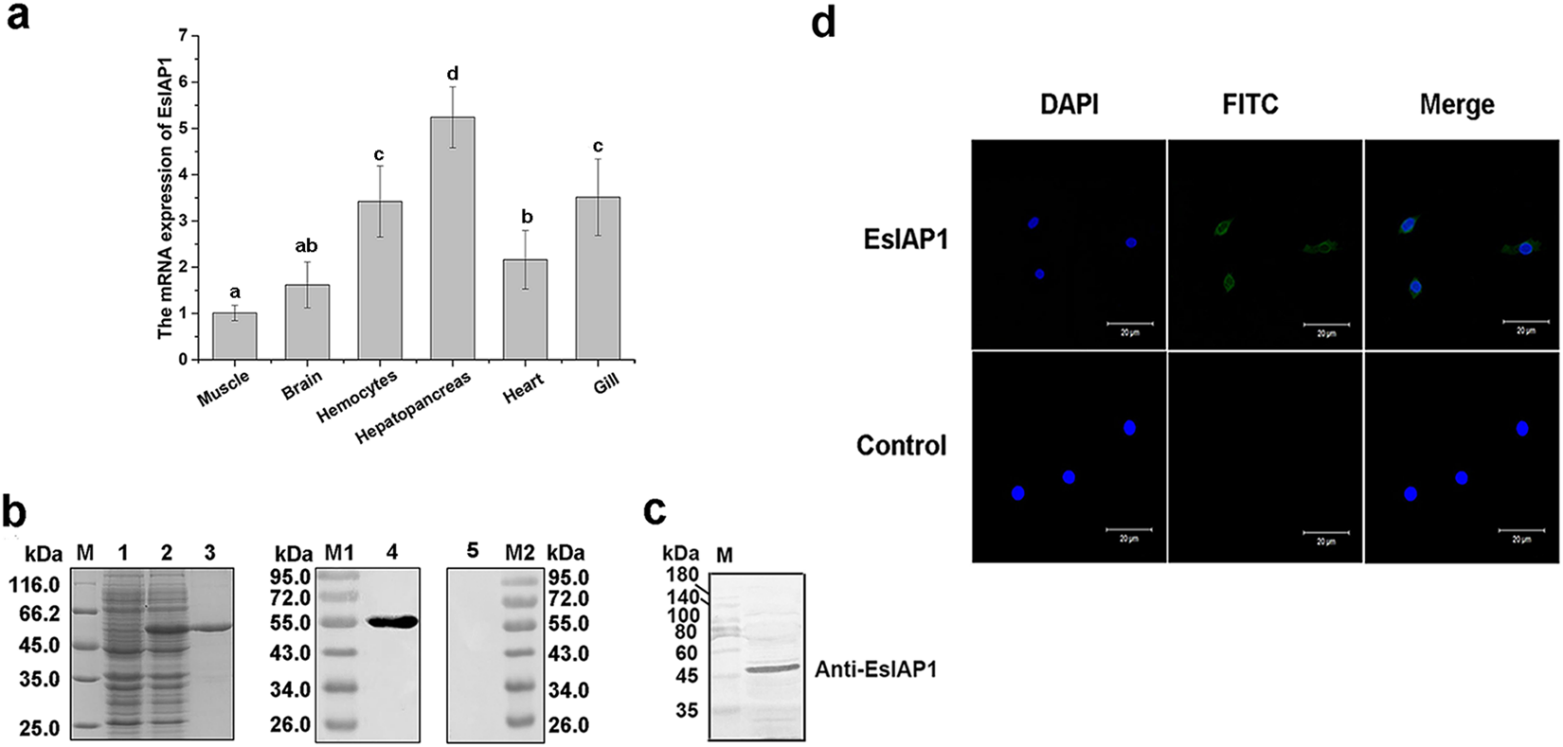
The mRNA expression of *Es*IAP1 in crabs and subcellular localization of *Es*IAP1 in hemocytes. (**a**) Quantitative real-time PCR (qRT-PCR) analysis of the expression level of *Es*IAP1 mRNA in different tissues. The different letters show that there exist significant differences comparing with other groups (*p* < 0.05). (**b**) SDS-PAGE and western blotting analysis of r*Es*IAP1. Lane M: protein marker; Lane 1: negative control for r*Es*IAP1 (without IPTG induction); Lane 2: IPTG induced r*Es*IAP1; Lane 3: purified r*Es*IAP1. Lane M1: protein marker; Lane 4: western blotting analysis of the r*Es*IAP1; Lane 5: western blotting analysis of the pre-immune serum from mice; Lane M2: protein marker. (**c**) The specific antibody detection of native *Es*IAP1. (**d**) Localization of *Es*IAP1 in hemocytes. Immunohistochemistry was performed to analyze the expression of *Es*IAP1 in hemocytes of *E. sinensis*. After incubation of polyclonal antibody of *Es*IAP1 or pre-immune serum (negative control), Alexa Fluor 488-labeled goat-anti-mouse antibody was used to detect *Es*IAP1. Nucleus was stained with DAPI (blue). Positive signals of *Es*IAP1 were shown in green. Scale bar = 20 μm. (For interpretation of the references to colour in this figure legend, the reader is referred to the web version of this article).

The mRNA transcripts of *Es*IAP1 could be detected in all the examined tissues, including hemocytes, hepatopancreas, heart, gill, brain and muscle with the highest expression level in hepatopancreas, which was 5.24-fold (*p* < 0.01) of that in muscle. The expression level of *Es*IAP1 mRNA in gill, hemocytes, heart and brain was 3.51-fold (*p* < 0.01), 3.41-fold (*p* < 0.01), 2.16-fold (*p* < 0.05) and 1.61-fold (*p* > 0.05) of that in muscle, respectively (Fig. 2a).

The purified r*Es*IAP1 was employed to prepare polyclonal antibody (Fig. 2b, Supplementary Fig. S1). A clear band with 55 kDa was revealed by western blotting assay, indicating the high recognition specificity of the polyclonal antibody against *Es*IAP1 (Fig. 2b, Supplementary Fig. S1). Pre-immune serum was used as negative control and no bands were detected (Fig. 2b, Supplementary Fig. S1). Western blotting assay of the native tissue sample with *Es*IAP1 antibody revealed that there was a distinct band of 50 kDa (Fig. 2c). Immunofluorescence assay was performed to detect the localization of *Es*IAP1 in hemocytes. The nucleus stained by DAPI was observed in blue, and the positive signal of *Es*IAP1 was in green. The positive fluorescence signals were mainly observed in the cytoplasm of hemocytes according to the merged pictures (Fig. 2d).

### The expression of *Es*IAP1 mRNA in hemocytes after LPS and *A. hydrophila* stimulation*s*

**Figure 3:**
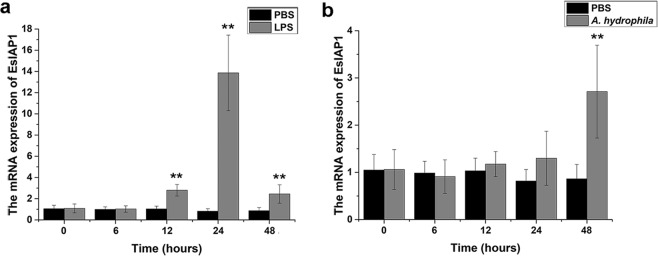
Temporal expression of the *Es*IAP1 transcripts in hemocytes after LPS and *A. hydrophila* stimulations. (**a**) qRT-PCR detection of the expressions of *Es*IAP1 in crabs challenged by LPS. (**b**) qRT-PCR detection of the expressions of *Es*IAP1 in crabs challenged by *A. hydrophila*.

The expression level of *Es*IAP1 mRNA in hemocytes increased significantly after the stimulations with LPS and *A. hydrophila*. After LPS stimulation, the mRNA transcripts of *Es*IAP1 increased significantly at 12 h (2.80-fold compared with that in PBS group, *p* < 0.01), reached the highest level of 13.86-fold (*p* < 0.01) at 24 h, and finally down-regulated to 2.44-fold (*p* < 0.01) at 48 h (Fig. 3a). After *A. hydrophila* stimulation, the relative expression level of *Es*IAP1 mRNA kept at quite low level and there was no significant difference from 0 to 24 h compared with that in PBS group. However, it increased significantly (2.71-fold of control group, *p* < 0.01) at 48 h post *A. hydrophila* stimulation (Fig. 3b).

### The mRNA expression of *Es*Caspase-3/7-1 and the activity of caspases in hemocytes after the gene silencing of *Es*IAP1

**Figure 4:**
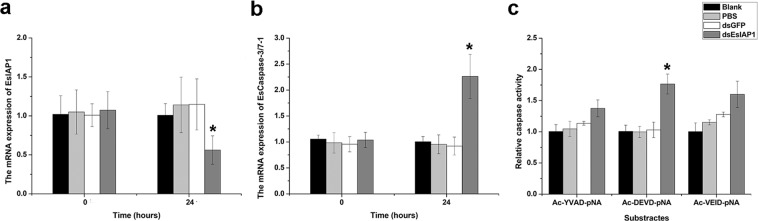
The mRNA and activity of caspase after the gene silencing of *Es*IAP1. (**a**) The expression level of *Es*IAP1 mRNA in hemocytes after gene silencing of *Es*IAP1. Comparison of the level of *Es*IAP1 was normalized to dsGFP group. (**b**) The expression level of the *Es*Caspase-3/7-1 mRNA in hemocytes of *Es*IAP1-interfered crabs. Comparison of the level of *Es*IAP1 was normalized to dsGFP group. (**c**) The activities of caspases were determined by measuring hydrolyzing activity against Ac-YVAD-*p*NA (substrate of caspase-1), Ac-DEVD-*p*NA (substrate of caspase-3) or Ac-VEID-*p*NA (substrate of caspase-6).

To further explore the function of *Es*IAP1 *in vivo*, the dsRNA-induced RNA interference (RNAi) was used to inhibit the expression of *Es*IAP1. The mRNA expression of *Es*IAP1 in hemocytes decreased to 0.47-fold (*p* < 0.05) (Fig. 4a), while the mRNA expression of *Es*Caspase-3/7-1 increased to 2.26-fold (*p* < 0.05) (Fig. 4b) at 24 h post the injection with sequence-specific dsRNA targeting *Es*IAP1 compared to that in dsGFP group. After *Es*IAP1 was silencd, the activity of caspase towards Ac-DEVD-pNA in hemocytes increased to 1.71-fold (*p* < 0.05) compared to that in the dsGFP group. While the activity of caspase-1 towards Ac-YAVD-pNA and caspase-6 towards Ac-VEID-pNA in hemocytes increased to 1.21-fold (*p* > 0.05) and 1.25-fold (*p* > 0.05) of that in the dsGFP group, respectively (Fig. 4c).

### The interation of r*Es*IAP1 with r*Es*Caspase-3/7-1 *in vitro*

**Figure 5:**
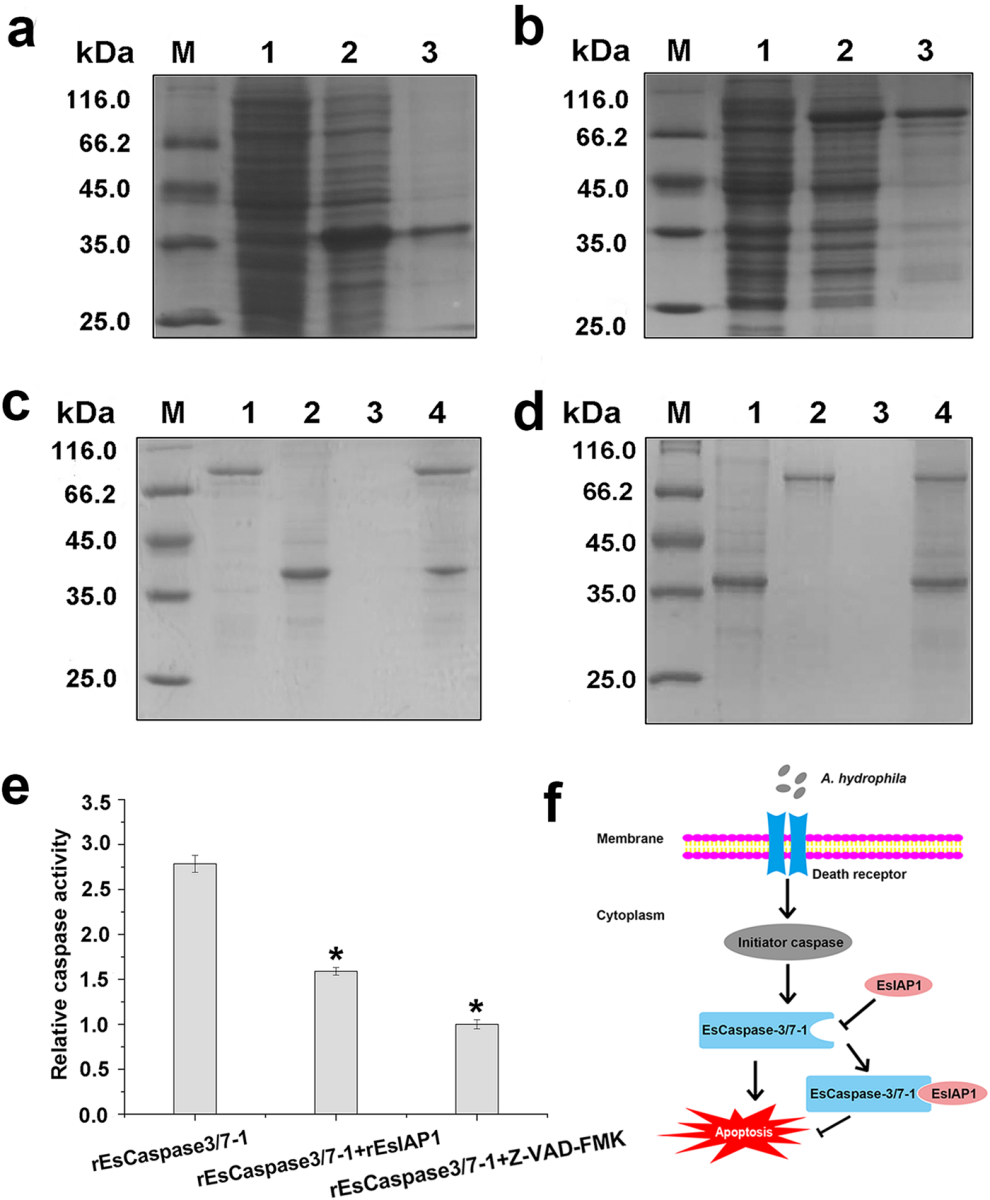
Interaction between *Es*IAP1 and *Es*Caspase-3/7-1 *in vitro*. (**a**) Purified r*Es*Caspase-3/7-1 (His). Lane 1: negative control for r*Es*Caspase-3/7-1 (His) (without IPTG induction); Lane 2: IPTG induced r*Es*Caspase-3/7-1 (His); Lane 3: purified r*Es*Caspase-3/7-1 (His). (**b**) Purified r*Es*IAP1 (GST). Lane 1: negative control for r*Es*IAP1 (GST, without IPTG induction); Lane 2: IPTG induced r*Es*IAP1 (GST); Lane 3: purified r*Es*IAP1 (GST). (**c**) Pull down by r*Es*IAP1 (GST). Lane 1: purified r*Es*IAP1 (GST); Lane 2: purified r*Es*Caspase-3/7-1 (His); Lane 3: washed liquid; Lane 4: eluted liquid. (**d**) Pull down by r*Es*Caspase-3/7-1 (His). Lane 1: purified r*Es*Caspase-3/7-1 (His); Lane 2: purified r*Es*IAP1 (GST); Lane 3: washed liquid; Lane 4: eluted liquid. (**e**) The activity of r*Es*IAP1 was detected with caspase-3 activity assay kit. (**f**) Model for *Es*IAP1 involvement in apoptosis pathway*. A. hydrophila* could activate caspase-mediated apoptosis pathway to initiate the activity of *Es*Caspase-3/7-1 to lead to hemocyte apoptosis. *Es*IAP1 could combine with *Es*Caspase-3/7-1 to inhibit the hemocyte apoptosis.

The interaction of *Es*IAP1 with *Es*Caspase-3/7-1 was analyzed by pull down assay to understand the regulatory mechanism of apoptosis. The full-length ORFs of *Es*IAP1 and *Es*Caspase-3/7-1 were expressed, and the purified r*Es*IAP1 and r*Es*Caspase-3/7-1 (Fig. 5a-b, Supplementary Fig. S2a-b) were used for GST and His pull down assays. Two distinct bands were observed in the elute liquid after pull down assay (Fig. 5c-d, Supplementary Fig. S2c-d). The results indicated that r*Es*IAP1 could directly combine and interact with r*Es*Caspase-3/7-1 *in vitro*.

### The hydrolytic activity of r*Es*Caspase-3/7-1 after incubation with r*Es*IAP1 *in vitro*

The hydrolyzing assay of caspase-3 substrate was employed to investigate the activity of r*Es*IAP1. The hydrolytic activity of r*Es*Caspase-3/7-1 was significantly inhibited after the incubation with r*Es*IAP1. r*Es*Caspase-3/7-1 displayed high hydrolytic activity towards Ac-DEVD-pNA (0.44 units per mg protein). After r*Es*Caspase-3/7-1 was incubated with r*Es*IAP1 or Z-VAD-FMK, the hydrolytic activities were 0.25 and 0.15 units per mg protein, respectively, which were significantly lower than that in r*Es*Caspase-3/7-1 group (*p* < 0.05) (Fig. 5e).

## Discussion

Apoptosis represents a fundamental biological process that relies on the activation of caspases^34^. IAPs are a family of negative regulators of both caspases and cell death^35^. In the present study, a novel IAP was identified from *E. sinensis* (designated *Es*IAP1). There were two BIR domains identified in *Es*IAP1, which was the typical domain of IAP family^6^. The deduced amino acid sequences of BIR1 and BIR2 domains in *Es*IAP1 shared high similarities (36.1%~53.5% and 45.9%~58.9%, respectively) with the corresponding domains of other IAP proteins (Fig. 1a,b). Moreover, the conserved spacing of cysteine and histidine residues (Cx_2_ Cx_6_ Wx_3_ Dx_5_ Hx_6_ C) in the other reported BIR2 domains were also found in *Es*IAP1, which was suggested to contribute to a novel zinc-binding fold^6^. These results suggested that *Es*IAP1 was a typical IAP family member. In the phylogenetic tree, *Es*IAP1 was firstly grouped with the crustacean IAPs to form a separated clade, then grouped with other arthropod IAP proteins, and finally clustered with vertebrate IAP proteins (Fig. 1c). These evidences collectively indicated that *Es*IAP1 belonged to the IAP family in crustaceans.

As regulators of the apoptotic machinery, IAPs play important roles in many physiological processes, including homeostasis maintenance, development of tissues, and immune responses^22,36,37^. In the present study, the mRNA transcripts of *Es*IAP1 could be detected in all the examined tissues inculding hemocytes, hepatopancreas, gill, muscle, brain, and heart (Fig. 2a). Similarly, the transcripts of *Cg*IAP2 were also detected in various tissues in oyster *C. gigas*^25^. The constitutive expression profile of *Es*IAP1 indicated that it might involve in many physiological processes of crabs. It has been reported that IAPs could regulate the activity of caspases, further modulate cell cycle proliferation and receptor-mediated signal transduction^9^. The higher level of *Es*IAP1 mRNA was observed in immune-associated tissues, including hemocytes, hepatopancreas and gill, which might be attributed to the cellular metabolism and innate immunity^38,39^. Crustacean hemocytes play important roles in the host immune response, including recognition, phagocytosis and cell communication^33,40,41^. Moreover, the high level of *Es*Caspase-3/7-1 and *Es*Caspase-3-like were observed in hemocytes^28,29^. The hemocytes were thus chosen as target to analyze the expression of *Es*IAP1. In the present study, the location of *Es*IAP1 in hemocytes was observed by immunofluorescence assay, and the positive signal was found to be mainly distributed in the cytoplasm, which was similar as the previous reports in other species^42–45^, possibly for the sake of binding to cytoplasmic caspase to regulate hemocyte apoptosis. LPS, a vital component of the outer wall of gram-negative bacteria, could trigger caspase-mediated apoptosis pathway^42,46^. The apoptosis pathway is regulated by initiator caspases (such as aspase-8 and caspase-10), which can be triggered by death receptor (like Fas, TNFR1 and TRAIL-R1/R2) to initiate the activity of effector caspases^43–45^. In the present study, the expression level of *Es*IAP1 mRNA was significantly up-regulated after LPS and *A. hydrophila* stimulations (Fig. 3a,b). It has been reported that apoptosis pathway could be activated after LPS and *A. hydrophila* stimulations in crustacean^47,48^. In *C. gigas*, *Cg*IAP2 was proposed to play a role in apoptosis inhibition in the immune defense against bacterial challenge^25^. Some crustacean IAPs such as *Pm*IAP and *Lv*IAP1 were suggested to be central to the regulation of hemocyte apoptosis^24,49^. Theses results suggested that *Es*IAP1 might exert important roles in immune defenses by regulating the apoptosis pathway in *E. sinensis*.

IAPs could regulate apoptosis through controlling caspase activities and caspase-activating platform formations, which also appeared to be important determinants of the responses of cells to endogenous or exogenous cellular injuries^13^. It was reported that c-IAP1 and c-IAP2 could directly bind to the activated caspase-3 and -7 to inhibit their activities in vertebrates^10^. In the present study, *Es*Caspase-3/7-1, an effector caspase, identified previously from *E. sinensis*^28^, were employed to investigate the binding activity of *Es*IAP1 with caspase. After r*Es*Caspase-3/7-1 was incubated with r*Es*IAP1 or Z-VAD-FMK, the hydrolyzing activity of r*Es*Caspase-3/7-1 was significantly decreased (Fig. 5e). This result was in coincidence with the observation that IAPs could inhibit the activation of effctor caspase^13,20^. The direct combination of r*Es*IAP1 with r*Es*Caspase-3/7-1 confirmed by pull down assay might explain the decrease of r*Es*Caspase-3/7-1 hydrolyzing activity after incubation with r*Es*IAP1 *in vitro*. These results suggested that r*Es*IAP1 could inhibit the hydrolytic activity of r*Es*Caspase-3/7-1 by interacting with r*Es*Caspase-3/7-1. Furthermore, the expression of *Es*Caspase-3/7-1 mRNA in hemocytes of crabs were significantly increased after the interference of *Es*IAP1, indicating the inhibitory regualtion of *Es*IAP1 on *Es*Caspase-3/7-1. The hydrolytic activity of caspase-3 was increased in hemocytes rather than caspase-1 and -6 after *Es*IAP1 was silenced. These results showed that *Es*IAP1 could regulate *Es*Caspase-3/7-1 and further inhibit hemocyte apoptosis *in vivo*. Similarly, the number of circulating hemocytes was increased in *Lv*IAP1-silenced shrimp because of the extensive apoptosis^24^. Some mammalian IAPs, such as c-IAP1 and c-IAP2, were also found to be involved in signaling cascades, and play important roles in TNF-induced apoptosis^50^. Therefore, it was speculated that *Es*IAP1 could inhibit apoptosis by regulating *Es*Caspase-3/7-1 in *E. sinensis*.

Caspases are activated to gain the full catalytic activity after being proteolytically cleaved to initiate apoptosis^51^. *Es*Caspase-3, -7 and -8 are characterized to play crucial roles in Cadmium-induced apoptosis^27^, and *Es*Caspase-3/7-1 and *Es*Caspase-3-like protein are found to be involved in innate immune response and induce apoptosis under pathogen stimulation^28,29^. IAPs inhibit such proteolytically activated caspases, and further regulate apoptosis^52^. The loss or inhibition of cIAP1, cIAP2 and XIAP causes the majority of cells to be sensitized to death receptor to induce cell death^53^. In *Drosophila*, DIAP1 normally inhibits both initiator and effector caspases^54,55^. In summary, this study suggested that LPS and bacterial challenge could activate the apoptosis pathway in *E. sinensis*. *Es*IAP1 could inhibit apoptosis by directly combining with *Es*Caspase-3/7-1 and inhibit its hydrolytic activity (Fig. 5f). These results provided novel idea to understand the modulatory role of IAP in apoptosis pathway in crustaceans.

## Materials and Methods

### Crabs, collection of tissues and immune stimulations

The crabs with an average weight of 20 g were collected from a commercial farm in Lianyungang, China, and cultured in aerated freshwater at 20 ± 2 °C for one week before processing^28^. Six crabs were sacrificed for determining the expression of *Es*IAP1 mRNA in different tissues. The tissues including muscle, heart, brain, hepatopancreas and gill were collected from crabs to detect the mRNA expression of *Es*IAP1 according to the previous study^56^. The hemolymph drawing from the last pair of walking legs from each crab by using a syringe was mixed with anticoagulant solution (510 mM NaCl, 100 mM glucose, 200 mM citric acid, 30 mM sodium citrate, 10 mM EDTA·2Na, pH 7.3) at a ratio of 1:1, and the hemocytes were harvested by centrifugation^57^. Tissues from two crabs were pooled together as one sample and there were three duplicates for each assay according to the previous methods^58^. The crabs were treated by the injections of 100 μL *Aeromonas hydrophila* (10^7^ CFU mL^−1^) and 100 μL lipopolysaccharide (500 μg mL^−1^) according to the previous reports^59^, respectively. Ninety crabs were employed and randomly divided into three groups. According to previous study, a volume of 100 μL alive *A. hydrophila* (1 × 10^7^ CFU mL^−1^) or lipopolysaccharide (LPS from *Escherichia coli* 0111:B4, L2630, Sigma Aldrich, USA; 100 µg mL^−1^) resuspending in PBS (40 mM NaCl, 2.7 mM KCl, 10 mM Na_2_HPO_4_, 2 mM KH_2_PO_4_, pH 7.4) was injected into the arthrodial membrane of the last pair of walking legs in the stimulation groups, respectively^28,59,60^. The crabs received an injection of 100 µL PBS were employed as control group. Six crabs were randomly sampled from each group at 0, 6, 12, 24 and 48 h after treatments.

### RNA extraction and cDNA synthesis

TRIzol reagent (Invitrogen) was uesd for the extraction of total RNA from *E. sinensis* tissue samples, and the first-strand cDNA was synthesised by using the PrimeScript™ real-time PCR kit (Takara, Japan) according to the manufacture’s instruction.

### Sequence analysis of *Es*IAP1

The sequence of IAP genes was analyzed by BLASTP (http://blast.ncbi.nlm.nih.gov/Blast.cgi) in the genome database (PRJNA305216) of *E. sinensis*^61^. The primers (*Es*IAP1-F and -R) were designed to clone the open reading frame (ORF) of *Es*IAP1. The multiple sequence alignments were created by Clustal W. The conserved domain was identified through the SMART (http://smart.embl-heidelberg.de/). MEGA6.0 package was used to construct phylogenetic tree.

### Purification of recombinant protein and preparation of polyclonal antibody

The full-length ORF sequences of *Es*IAP1 and *Es*Caspase-3/7-1 were amplified with specific primers (r*Es*IAP1-His-F and -R, r*Es*Caspase-3/7-1-F and -R) (Table 1). The PCR products were inserted into pET-22b vector (Novagen) with a His-tag. r*Es*IAP1-GST-F and -R (Table 1) were used to amplify *Es*IAP1, and the PCR products were inserted into the pGEX4T-1 vector (GE Healthcare) with a GST-tag. All those recombinant plasmids were transformed into *E. coli* BL21 (DE3) competent cells. These prokaryotic proteins were purified by a Ni^2+^ chelating sepharose column or GST-resin, following the manufacturers’ instructions. Their concentrations were measured by BCA kit (Beyotime). The preparation of antiserum was performed as previously described^62^.

**Table 1:** Primers used in this study.

Primers	Sequence (5′-3′)
*Es*IAP1-F	ATGGACATGTCTCGTCGGCAGTT
*Es*IAP1-R	TCAGCCGATGATGGGCCG
r*Es*Caspsase-3/7-1-F	GGGAATTCCATATGGATAACATCAAGGAAAATGG
r*Es*Caspsase-3/7-1-R	CCGCTCGAGATACTTGGGAGACAGGAAGACCT
r*Es*IAP1-F (His)	GGAATTCCATATGGACATGTCTCGGCAGTT
r*Es*IAP1-R (His)	CCGCTCGAGGCCGATGATGGGCCG
r*Es*IAP1-F(GST)	CGCGGATCCATGGACATGTCTCGGCAGTT
*Es*IAP1-R(GST)	ACGCGTCGACGCCGATGATGGGCCG
*Es*IAP1-RNAi-F	TAATACGACTCACTATAGGGATGGACATGTCTCGTCGGCAGTT
*Es*IAP1-RNAi-R	TAATACGACTCACTATAGGGGCCGATGATGGGCCG
GFP-RNAi-F	TAATACGACTCACTATAGGGCGACGTAAACGGCCACAAGT
GFP-RNAi-R	TAATACGACTCACTATAGGGCTTGTACAGCTCGTCCATGC
*Es*IAP1-qRT-F	CGCCAGGGTTTTCCCAGTCACGAC
*Es*IAP1-qRT-R	CATCAAGGAGAAACTGTGCT
*Es*Caspsase-3/7-1-qRT-F	CCACCACTGCTGACTTCTTGATA
*Es*Caspsase-3/7-1-qRT-R	AGACAGGAAGACCTTTCTCATCAA
*Es*-β-Actin-F	CCCATCTACGAGGGCTACGC
*Es*-β-Actin-R	CCTTGATGTCTCGCACGATTTCT

### Western blotting and immunohistochemistry analysis of *Es*IAP1

The western blotting assay was performed according to the previous report^28^. Recombinant protein was separated by SDS-PAGE, and transferred onto nitrocellulose membrane. After blocking for 1 h with 5% non-fat milk in TBST, the membrane was incubated successively with 1/1000 diluted poly-antibody of anti-*Es*IAP1 as first antibody and 1/10,000 diluted goat-anti-mouse IgG (Sangon) as secondary antibody. After washing in TBST, the membrane dipped in ECL substrate system (Thermo Scientific) for 2 min, then imaged by Amersham Imager 600 (General Electric Company).

The hemocytes were resuspended with DMEM (Sangon) and then added into poly-L-lysine pre-coated dishes. After fixed with 4% paraformaldehyde (PFA, diluted in PBS), the dishes were blocked with 3% fetal bovine serum albumin (diluted in PBS) at 37 °C for 30 min, followed by washing with PBST (PBS with 0.1% tween-20). The dishes were then successively incubated with 1/1000 diluted anti-*Es*IAP1 antibody at 37 °C for 1 h and 1/1,000 diluted Alexa Fluor 488-labeled goat-anti-mouse antibody at 37 °C for 1 h. After final washing with PBST, DAPI (1 µg/mL in PBS) was used to stain the nucleus, and the dishes were observed by fluorescence microscope (ZEISS).

### RNA interference

The RNA interference assay of *Es*IAP1 was performed according to the previous report^32^. T7 promoter linked primers (GFP-RNAi-F and -R, *Es*IAP1-RNAi-F and -R) were used to amplify the cDNA sequence of dsGFP (657 bp) and ds*Es*IAP1 (1,356 bp), respectively. The dsRNAs of *Es*IAP1 and GFP were diluted with PBS to the final concentration of 0.5 μg μL^−1^. The crabs were treated by the injections with 100 μL PBS, dsGFP and ds*Es*IAP1, respectively. The untreated crabs were used as blank group. Six crabs from each group were randomly sampled at 0 and 24 h post injections. The hemocytes were divided into two parts, and one aliquot of hemocyte sample was used to estimate the silencing efficiency, while the other was used for the measurment of caspase activity.

### Quantitative real-time PCR (qRT-PCR) analysis of mRNA expression

qRT-PCR was conducted according the previous reports^63^. Two primers, *Es*IAP1-qRT-F and -R, were used in qRT-PCR to detect the expression of *Es*IAP1. The fragment amplified by primers of *Es-*β-actin (*Es*-β-Actin-F and -R) were employed as reference. The gene expression analysis was performed using the 2^−∆∆Ct^ method^63^, and all data were given in terms of relative mRNA expression of mean ± S.E. (N = 3).

### Pull down assay

The pull down assay was carried out according to the previous report^63^. The proteins of r*Es*IAP1 (GST) and r*Es*caspase-3/7-1 (His) (30 μg) were mixed with 20 μL of glutathione resin (for GST-tagged proteins) or charged nickel-nitrilotriacetic acid beads (for His-tagged proteins), respectively. The mixture (resin and binding proteins) was incubated at room temperature for 2 h with slight rotation, and then washed for three times by centrifuging at 500 × *g* for 3 min to remove the unbound proteins. The tested protein (r*Es*Caspase-3/7-1-His and r*Es*IAP1-GST), without GST tag or His tag, was added into the mixture containing the nickel-nitrilotriacetic acid beads or glutathione resin, and gently rotated at room temperature for 2 h. After washing three times, the mixture was analyzed by SDS-PAGE.

### The hydrolyzing function assays of r*Es*IAP1 *in vitro*

The potential inhibiting hydrolytic activity of r*Es*IAP1 was detected using the caspase-3 activity assay kit (Beyotime) under the manufacturer’s manual^10^. The protein concentration of purified r*Es*IAP1-His and r*Es*Caspase-3/7-1 was adjusted to 1 mg mL^−1^. There were three experimental grous, including blank group (r*Es*Caspase-3/7-1), r*Es*IAP1 group (r*Es*IAP1 + r*Es*Caspase-3/7-1), and Z-VAD-FMK (pan caspase inhibitor) group (Z-VAD-FMK + r*Es*Caspase-3/7-1). r*Es*Caspase-3/7-1 protein in r*Es*IAP1 and Z-VAD-FMK groups were pre-incubated with r*Es*IAP1 and Z-VAD-FMK at final concentrations of 100 μg mL^−1^ and 100 μM, respectively^64^. The mixtures were incubated at 37 °C for 1 h and absorbance value was monitored at 405 nm by the SpectraMax 190 (Molecular Devices, Sunnyvale, CA, USA). The blank group (r*Es*Caspase-3/7-1) was employed as the control, and the hydrolytic activity of r*Es*IAP1 was determined by comparing the hydrolytic activity of r*Es*Caspase-3/7-1 against Ac-DEVD-*p*NA.

### Hydrolyzing activity analysis of caspases in *Es*IAP1-interfered crabs

The hydrolyzing activity of caspases in hemocytes was examined according to the method described by previous study^29^. The hydrolytic activity of the crab hemocyte protein was detected at 0 and 24 h after the injection of *Es*IAP1-dsRNA. The protein concentration of the supernatant was measured using the Bradford Protein Assay Kit (Beyotime) and adjusted to 1 mg mL^−1^ with lysate buffer. The hydrolytic activity of caspases was examined with the substrate Ac-YAVD-pNA, Ac-DEVD-*p*NA and Ac-VEID-pNA using the caspase-1, -3 and -6 activity assay kit (Beyotime, Shanghai, China) under the manufacturer’s manual. The absorbance values of the reaction mixture was monitored at 405 nm using Spectra Max 190 (Molecular Devices, Sunnyvale, CA, USA). The different absorbance values represented the cleavage and release of *p*NA. The blank group was used as the reference.

### Statistical analysis

The data (represented as mean ± S.E., N = 3) were calculated by using the 2^−∆∆Ct^ method^65^, and analyzed with *t*-test. Significant differences across controls were indicated with an asterisk at *p* < 0.05, and two asterisks at *p* < 0.01.

## Supplementary information

Supplementary information.
