# Supplementary material for: An inhibitor of apoptosis protein (EsIAP1) from Chinese mitten crab Eriocheir sinensis regulates apoptosis through inhibiting the activity of EsCaspase-3/7-1
Source: Sci Rep. 2019 Dec 31;9:20421. doi: 10.1038/s41598-019-56971-1 (PMC6938513; doi:10.1038/s41598-019-56971-1)
Supplement: Supplementary file 1 — Supplementary information. [file 41598_2019_56971_MOESM1_ESM.pdf]

Supplementary information

An inhibitor of apoptosis protein (*Es*IAP1) from Chinese mitten crab *Eriocheir sinensis* regulates apoptosis through inhibiting the activity of *Es*Caspase-3/7-1

Chen Qu[a], Jiejie Sun[a], Qingsong Xu[a,d], Xiaojing Lv[a,b,c,d],Wen Yang[a], Feifei Wang[a], Ying Wang[a], Qilin Yi[a,c,d], Zhihao Jia[b], Lingling Wang[a,b,c,d], Linsheng Song [a,b,c,d]*

[a] Liaoning Key Laboratory of Marine Animal Immunology, Dalian Ocean University, Dalian 116023, China

[b] Laboratory of Marine Fisheries Science and Food Production Processes, Qingdao National Laboratory for Marine Science and Technology, Qingdao 266235, China

[c] Liaoning Key Laboratory of Marine Animal Immunology & Disease Control, Dalian Ocean University, Dalian 116023, China

[d] Dalian Key Laboratory of Aquatic Animal Disease Prevention and Control, Dalian Ocean University, Dalian 116023, China

Full-length SDS-PAGE gels and blots images for Figure 2b-c

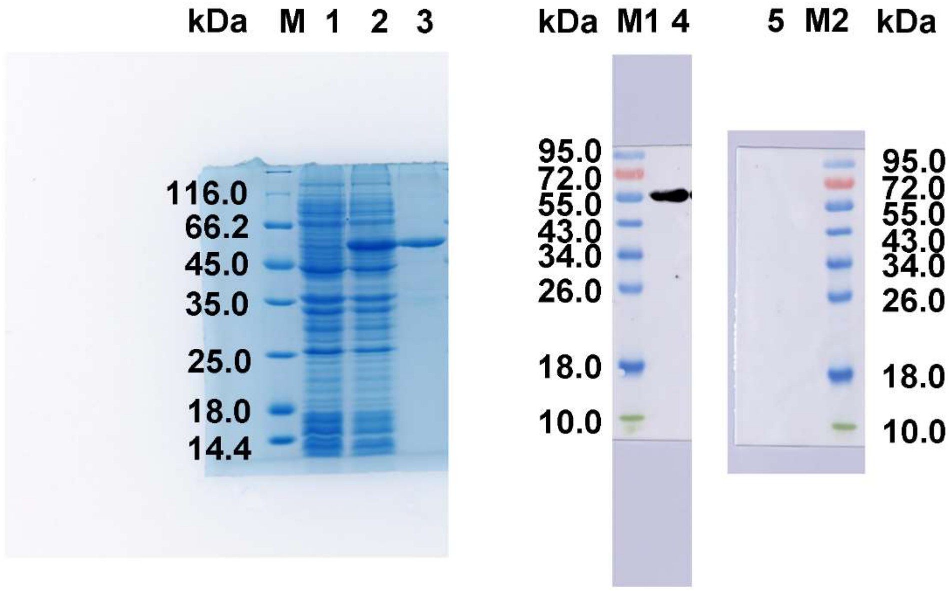

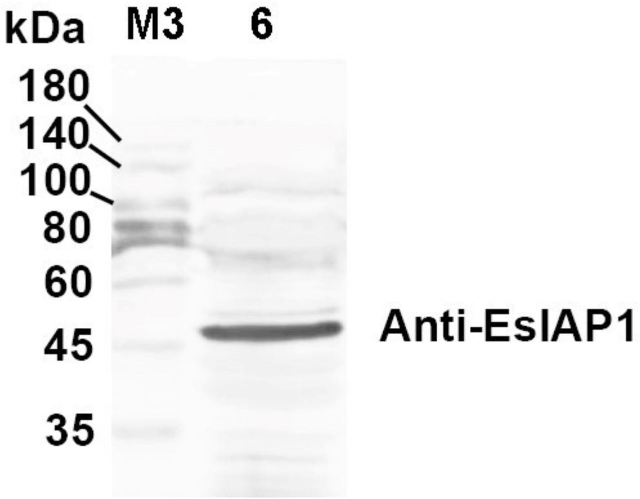

SDS-PAGE and western blotting analysis of *Es*IAP1. Lane M: protein marker; Lane 1: negative control for r*Es*IAP1 (without IPTG induction); Lane 2: IPTG induced r*Es*IAP1; Lane 3: purified r*Es*IAP1. Lane M1: protein marker; Lane 4: western blotting analysis of the r*Es*IAP1; Lane 5: western blotting analysis of the pre-immune serum from mice; Lane M2: protein marker.; Lane M3: protein marker; Lane 6: the specific antibody detection of *Es*IAP1.

Full-length SDS-PAGE gels images for Figure 5a-b

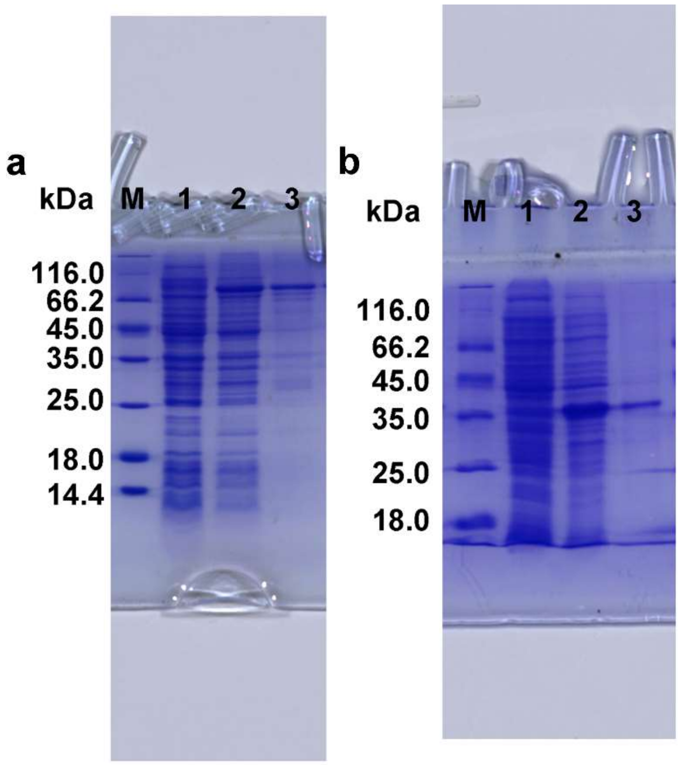

(a) Purified r*Es*Caspase-3/7-1 (His). Lane 1: negative control for r*Es*Caspase-3/7-1 (His) (without IPTG induction); Lane 2: IPTG induced r*Es*Caspase-3/7-1 (His); Lane 3: purified r*Es*Caspase-3/7-1 (His). (b) Purified r*Es*IAP1 (GST). Lane 1: negative control for r*Es*IAP1 (GST, without IPTG induction); Lane 2: IPTG induced r*Es*IAP1 (GST); Lane 3: purified r*Es*IAP1 (GST).

Full-length SDS-PAGE gels images for Figure 5c-d

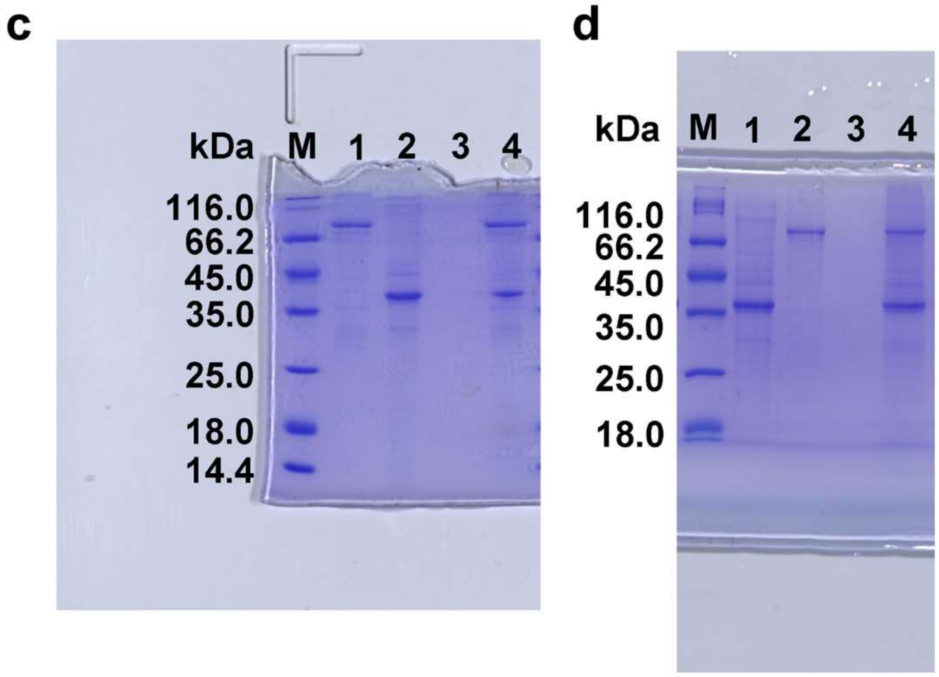

(c) Pull down by r*Es*IAP1 (GST). Lane 1: purified r*Es*IAP1 (GST); Lane 2: purified r*Es*Caspase-3/7-1 (His); Lane 3: washed liquid; Lane 4: eluted liquid. (d) Pull down by r*Es*Caspase-3/7-1 (His). Lane 1: purified r*Es*Caspase-3/7-1 (His); Lane 2: purified r*Es*IAP1 (GST); Lane 3: washed liquid; Lane 4: eluted liquid.
